# Electroanalysis of Ibuprofen and Its Interaction with Bovine Serum Albumin

**DOI:** 10.3390/molecules28010049

**Published:** 2022-12-21

**Authors:** Muhammad Dilshad, Afzal Shah, Shamsa Munir

**Affiliations:** 1 Department of Chemistry, Quaid-Azam University, Islamabad 45320, Pakistan; 2 School of Applied Sciences and Humanities, National University of Technology (NUTECH), Islamabad 44000, Pakistan

**Keywords:** modified glassy carbon, ibuprofen, bovine serum albumin, inflammation

## Abstract

The current work presents a sensitive, selective, cost-effective, and environmentally benign protocol for the detection of ibuprofen (IBP) by an electrochemical probe made of a glassy carbon electrode modified with Ag-ZnO and MWCNTs. Under optimized conditions, the designed sensing platform was found to sense IBP up to a 28 nM limit of detection. The interaction of IBP with bovine serum albumin (BSA) was investigated by differential pulse voltammetry. IBP−BSA binding parameters such as the binding constant and the stoichiometry of complexation were calculated. The results revealed that IBP and BSA form a single strong complex with a binding constant value of 8.7 × 10^13^. To the best of our knowledge, this is the first example that reports not only IBP detection but also its BSA complexation.

## 1. Introduction

**Scheme 1:**
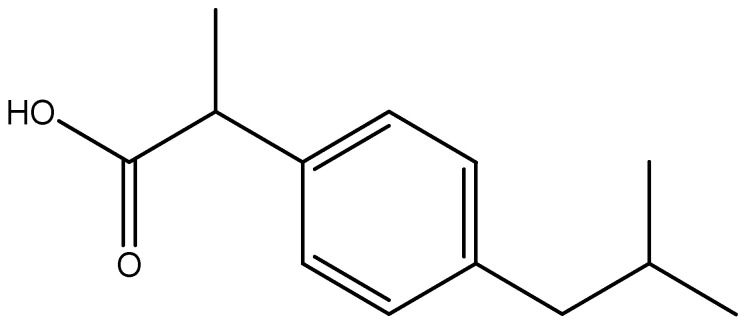
Chemical structure of ibuprofen.

Drugs facilitate the prevention and cure of diseases by strengthening the immune system. The mechanism of drug action is a specific biochemical interaction that results in targeted pharmacological effect. This action includes binding of the drug molecule to a specific targeted biological species such as enzymes or receptors [1,2,3,4]. Overdosage of drugs can result in adverse short-term or long-term health effects [5]. Drugs affect or alter the physiology of living organisms [6]. They stimulate a biological reaction by targeting macromolecules in the body [7]. As a rule, most drugs impede a particular biological response by interfering with the neurological system (particularly the brain). Based on pharmacodynamics, drugs can be classified as depressants, hallucinogens, and stimulants. Drugs can also be classified into analgesics and therapeutics. The current work presents electroanalysis of a non-steroidal anti-inflammatory drug (NSAID), ibuprofen (Scheme 1), which is the third most popular, prescribed, and sold-over-the-counter drug in the world [8]. The World Health Organization has listed ibuprofen (IBP) as an “essential drug” [9]. It is extensively used as a pain reliever in conditions such as menstrual cramps, headaches, arthritis, and a wide variety of other common aches and pains [10,11]. It plays an anti-inflammatory role by prohibiting the production of pro-inflammatory prostaglandins through inhibition of the enzyme cyclooxygenase [12].

IBP enters into the environment due to improper pharmaceutical disposal during treatment. IBP manufacturing industries are a major contributor to the entrance of this drug into bodies of water. The sources of IBP water pollution can be seen in Figure S1. The toxicity of IBP metabolites exceeds that of the parent molecule. After excretion, IBP makes its way into wastewater treatment plants, sewage treatment plants, rivers, lakes, groundwater, soil, etc. A number of methods have been proposed for determining the concentration and effects of IBP in aquatic organisms [13]. To examine the short-term and long-term effects of IBP exposure, toxicity tests have been conducted on water-dwelling species. IBP is an emerging organic contaminant as its risk quotient is quite high [14]. Hence, part of the current work is focused on designing a sensitive electrochemical platform for the detection of IBP.

**Figure 1:**
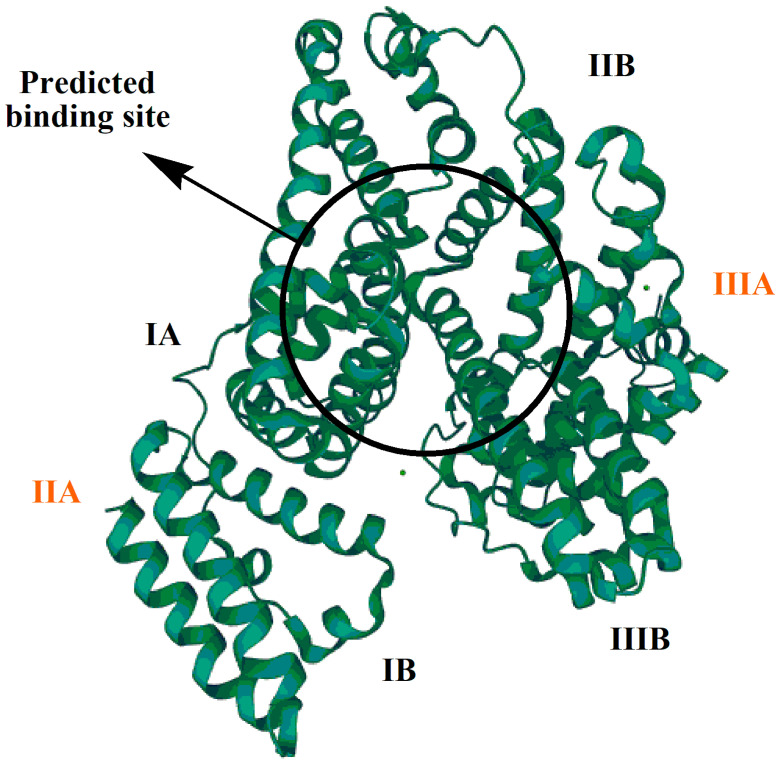
Bovine serum albumin (BSA).

The protein–drug binding process involves complexation of a drug with protein. Protein–drug binding can be intracellular or extracellular. Intracellular binding involves the drug’s interaction with cell proteins, eliciting a pharmacological response; the receptors with which the drug interacts are known as primary receptors. Extracellular binding does not usually result in a pharmacological response, and such drug receptors are known as secondary or silent receptors. The nature of a drug binding to a protein can be reversible or irreversible [15]. IBP pharmacokinetically interacts with BSA, which is a natural and very abundant (59%) plasma protein. BSA has a high affinity for binding with drug ligands and metabolites [16]. As shown in Figure 1, BSA has three domains (I, II, and III) and two sub-domains (A and B). The predicted drug-binding site is present in the sub-domains II A and III A.

Consider albumin (ALB) as the host protein that forms a complex with the guest drug (D). The binding constant ($\beta_{s})$, also known as the affinity constant or association constant, is determined from the stoichiometry of complexation between ALB and D according to the following equation [17]:

(1) $$\mathrm{ALB}+\mathrm{mD}\overset{\beta_{s}}{\Leftrightarrow }\;\mathrm{ALB}-\mathrm{mD}\;$$

Here ALB−mD represents the protein drug complex, and the stoichiometric co-efficient m indicates the number of drug molecules interacting per single molecule of protein. [FD] is the concentration of the free drug. The square brackets indicate the concentration of that particular species in Equation (2).

At equilibrium, $\beta_{s}$ can be obtained by:

(2) $$\beta_{s}=\frac{\left[\mathrm{ALB}-\mathrm{mD}\right]}{\left[\mathrm{ALB}\right]{\left[\mathrm{FD}\right]}^{m}}$$

Electrochemical study shows that by adding albumin to a constant volume of drug solution, the peak current of the drug decreases owing to its interaction with the albumin. The maximum decrease in peak current occurs when the maximum amount of a drug interacts with the protein. The decrease in peak current occurs due to slow mobility of the drug–protein complex as compared to the free drug. Hence, involvement of more drug molecules in interaction with proteins results in a smaller amount of the free drug concentration in solution. Moreover, a greater concentration of albumin leads to a maximum decrease in peak current intensity due to the formation of a greater number of complex molecules. Mathematically:

$${\Delta I}_{\max}\propto \left[\mathrm{ALB}\right]\mathrm{total}$$

$${\Delta I}_{\max}=K\;\left[\mathrm{ALB}\right]\mathrm{total}$$

where K is the proportionality constant

(3) ΔI=K [ALB−mD]

(4) $${\Delta I}_{\max}-\Delta I=K\left[\left[\mathrm{ALB}\right]\mathrm{total}-\left(\mathrm{ALB}-\mathrm{mD}\right)\right]$$

As

[ALB]total=[ALB]+[ALB−mD]

By substituting the value of [ALB]_total_, one obtains the following equation:

(5) $${\Delta I}_{\max}-\Delta I=K\left[\mathrm{ALB}\right]$$

$$\frac{\Delta I}{{\Delta I}_{\max}-\Delta I}=\frac{\left[\mathrm{ALB}-\mathrm{mD}\right]}{\left[\mathrm{ALB}\right]}$$

The right side of the above equation is equal to $\beta_{s}{\left[\mathrm{FD}\;\right]}^{m}$ according to Equation (2). Hence,

$$\frac{\Delta I}{{\Delta I}_{\max}-\Delta I}=\beta_{s}{\left[\mathrm{FD}\right]}^{m}$$

$$\mathrm{Log}\left(\frac{\Delta I}{{\Delta I}_{\max}-\Delta I}\right)=\mathrm{Log}\;\left(\beta_{s}{\left[\mathrm{FD}\;\right]}^{m}\right)$$

(6) $$\mathrm{Log}\left(\frac{\Delta I}{{\Delta I}_{\max}-\Delta I}\right)={\mathrm{Log}\;\beta }_{s}+m\;\mathrm{Log}\;\left[\mathrm{FD}\right]$$

A linear relationship between $\mathrm{Log}\left(\frac{\Delta I}{{\Delta I}_{\max}-\Delta I}\right)$ and Log [FD] indicates the formation of a single drug–protein complex. On the other hand, a non-linear relationship would suggest the formation of multiple complexes with different stoichiometry. If the Φ = $\;\mathrm{Log}\left(\frac{\Delta I}{{\Delta I}_{\max}-\Delta I}\right)$ and Log [FD] plot shows two linear segments, then two types of complexes have been formed, and this will result in slopes m_1_ and m_2_. When multiple complexes are formed, then the following equation can be used:

(7) $$\Delta I=\frac{\Delta I_{1}\beta_{1}\left[\mathrm{FD}\right]+\Delta I_{2}\beta_{2}\;{\left[\mathrm{FD}\right]}^{2}+\cdots +\Delta I_{n}\beta_{n}{\left[\mathrm{FD}\right]}^{n}}{1+\beta_{1}\left[\mathrm{FD}\right]+{\;\beta }_{2}\;{\left[\mathrm{FD}\right]}^{2}+\cdots +{\;\beta }_{n}{\left[\mathrm{FD}\right]}^{n}}$$

where ΔI is the total decrease of peak current *I*_p_ obtained through a current voltage measurement.

$$f_{1}=\frac{\Delta I}{\left[\;\mathrm{FD}\;\right]}$$

(8) $$f_{1}=\frac{\Delta I_{1}\beta_{1}+\Delta I_{2}\beta_{2}\;{\left[\mathrm{FD}\right]}^{1}+\cdots +\Delta I_{n}\beta_{n}{\left[\mathrm{FD}\right]}^{n-1}}{1+\beta_{1}\left[\mathrm{FD}\right]+{\;\beta }_{2}\;{\left[\mathrm{FD}\right]}^{2}+\cdots +{\;\beta }_{n}{\left[\mathrm{FD}\right]}^{n}}$$

The binding constants obtained in the case of multiple types of complex formation can be discovered using detailed equations given in the Supporting Information File.

All the above mentioned equations for binding constant determination involve calculation from peak current of the drug in the presence of protein. In this regard, electroanalytical techniques are the most promising options. The importance of the detection of drugs and their metabolites using an electrochemical platform for safeguarding the health of patients has pushed researchers to develop sophisticated electroanalytical tools for their monitoring [18]. The detection method should possess the qualities of high selectivity and sensitivity so that it can be effective for sensing biotoxins [19]. In this regard, the current research work aims to design a modified glassy carbon electrode using a composite of multiwalled carbon nanotubes and Ag-doped ZnO (MWCNTs/Ag-ZnO) as an electrode modifier for the trace-level detection of IBP. Ag-ZnO was selected as it is an effective nanomaterial that exhibits electro-inactivity in the chosen potential window and its integration with MWCNTs results in significantly enhanced performance owing to the decrease in interfacial charge transfer resistance by producing a number of active sites for the adsorption of more analyte molecules and consequent intense current signal. Moreover, components of this modifier are environmentally benign.

## 2. Results and Discussion

### 2.1. Material Characterization

**Figure 2:**
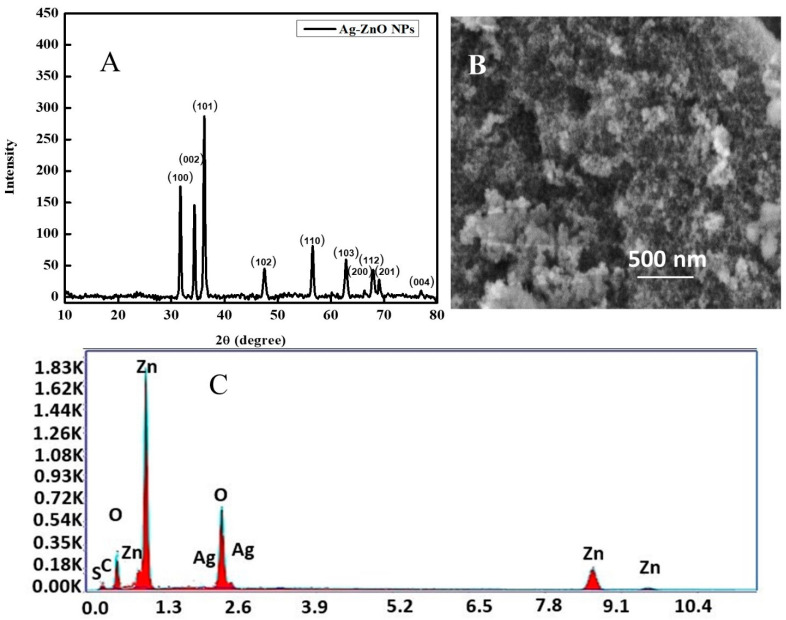
(**A**) X-ray diffractogram of Ag-ZnO, (**B**) SEM micrograph of Ag-ZnO, and (**C**) EDX spectrum of Ag-ZnO nanoparticles.

The synthesized Ag-ZnO nanoparticles were qualitatively characterized using X-ray diffraction analysis (XRD). The XRD diffractogram obtained for the synthesized nanoparticles is shown in Figure 2A. The peaks positioned at 31.75°, 34.37°, 36.15°, 47.55°, 56.48°, 62.81°, 66.37°, 67.90°, 69.12°, and 76.96° 2-theta values corresponded to the (100), (002), (101), (102), (110), (103), (200), (112), (201), and (004) diffraction planes, respectively. The X-ray diffractogram was in good agreement with the standard diffraction pattern of ZnO obtained from JCPDS card no. 36-1451.

SEM analysis of the surface characteristics of the produced Ag-ZnO revealed some interesting results. As synthesized, Ag-doped ZnO was visualized using an SEM micrograph (Figure 2B) with an accelerating voltage of 20 kV. Aggregation is indicated by the SEM micrograph, which reveals nanoparticles of varying sizes that have clumped together. The size can vary from one sample to the next depending on the condition of the precursors and the processes that were employed to synthesize the compound. The SEM shows the co-existence of smaller and larger nanoparticles. Agglomeration of smaller particles could be responsible for the formation of larger nanoparticles, and this phenomenon also explains why the forms of individual nanoparticles are obscured. The growing van der Waals forces, also known as intermolecular forces, between the silver nanoparticles and the zinc oxide network matrix caused the nanoparticles to become agglomerated. SEM studies indicated the particle size range at 11.7–20.8 nm, yet a large population of particles smaller than 11.7 nm is evident in the micrograph. The elemental composition of the synthesized material was analyzed using energy dispersive X-ray spectroscopy (EDX). The different elemental peaks such as Ag, Zn, O, S, and C were obtained as shown in Figure 2C. According to EDX analysis, the ZnO NPs with Ag doping were predominantly made up of Zn and O, with some trace amounts of Ag. Minor traces of S emerge from chemical impurities, whereas the presence of C originates from the carbon tape employed in the SEM analysis.

### 2.2. Electrochemical Characterization of IBP Using the Designed Sensor

The developed sensing platform was electrochemically characterized using cyclic voltammetry and electrochemical impedance spectroscopy. The surface area of the working electrode is a crucial aspect that has a considerable influence on the working ability of the electrochemical sensing platform. The cyclic voltammetric experiment was carried out to investigate the electroactive surface area of electrodes in a 5 mM solution of K_3_[Fe(CN)_6_] (redox probe) in 0.1 M KCl (supporting electrolyte). The current response of [Fe(CN)_6_]^3/4−^ for bare, Ag-ZnO-, MWCNT-, and MWCNT/ZnO-modified GCEs were investigated.

**Figure 3:**
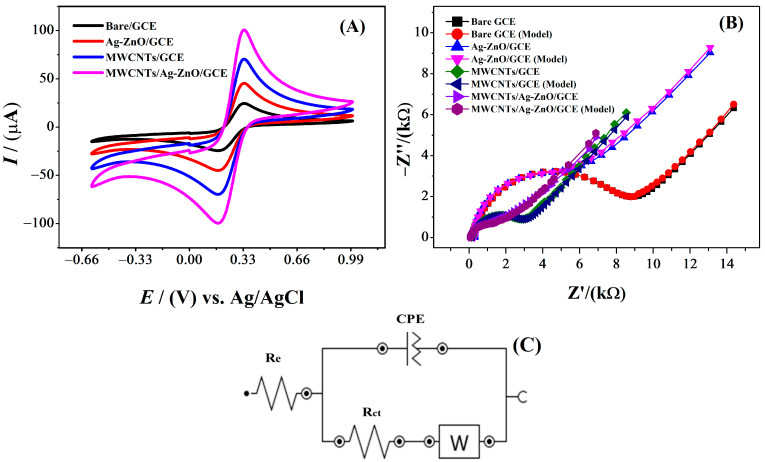
(A) Cyclic voltammograms for bare and modified glassy carbon electrodes recorded in 5 mM K_3_[Fe(CN_6_)] with 0.1 M KCl as supporting electrolyte at a scan rate of 100 mVs^−1^ (**B**) Nyquist plots of bare and modified GCEs in a solution of 5 mM K_3_[Fe (CN)_6_] as a redox probe and 0.1 M KCl as a supporting electrolyte, and (**C**) equivalent circuit corresponding to EIS data.

Figure 3A shows the cyclic voltammograms for 5 mM [Fe(CN)_6_]^4−^ obtained for the modified GCEs in 0.1 M KCl supporting electrolyte. In these voltammograms, the current peak at 0.34 V corresponds to the oxidation of [Fe(CN)_6_]^4−^ to [Fe(CN)_6_]^3−^, while the current peak at 0.21 V corresponds to the reduction of [Fe(CN)_6_]^3−^ to [Fe(CN)_6_]^4−^. From these voltammograms, the peak currents were found to be 100, 70, 48, and 25 μA for the MWCNT/Ag-AnO/GCE, MWCNT/GCE, Ag-ZnO/GCE, and bare GCE, respectively. The current values were employed to calculate the electroactive surface area, and the Randles–Sevcik equation was utilized for both unmodified and modified electrodes [20].

(9) $$I_{p}=2.69\times 10^{5}\;n^{\frac{3}{2}}\;AD^{\frac{1}{2}}\;v^{\frac{1}{2}}\;C\;$$

where *I*_p_ represents the anodic peak current in amperes, *D* represents the diffusion coefficient of the analyte in cm^2^ s^−1^, A is the electroactive surface area in cm^2^, *v *is the scan rate with a potential scan rate of V s^−1^, *C* represents the concentration of the probe in mol cm^−3^, and *n* represents the number of electrons.

For [K_3_Fe(CN)_6_], *D* = 7.6 × 10^6^ cm^2^s^−1^ and *n* = 1. Table S1 demonstrates the electroactive surface areas of the GCE, Ag-ZnO/GCE, MWCNTs/GCE, and MWCNTs/Ag-ZnO/GCE. In comparison to the active surface area of the bare electrode (0.02 cm^2^), the active surface area of the MWCNTs/Ag-ZnO/GCE (0.09 cm^2^) was nearly 4.5 times greater. Figure 3A shows cyclic voltammograms for 5 mM [Fe(CN)_6_]^3−^ obtained for the modified GCEs in 0.1 M KCl supporting electrolyte. In these voltammograms, the redox couple corresponding to the oxidation of [Fe(CN)_6_]^4−^ to [Fe(CN)_6_]^3−^ and reduction of [Fe(CN)_6_]^3−^ to [Fe(CN)_6_]^4−^ are observable at 0.33 V and 0.17 V, respectively. An obvious twofold, threefold, and fourfold increase in peak current can be seen for the Ag-ZnO/GCE, MWCNTs/GCE, and MWCNTs/Ag-ZnO/GCE, respectively. The faster electron transport of the redox probe can be related to better conductivity and more active sites provided by the modifier components at the GCE surface.

The method of electrochemical impedance spectroscopy did not impart any damage to the tested material. Through EIS, charge transfer kinetics for both bare and modified GCEs were investigated in a 5 mM solution of K_3_[Fe(CN)_6_] in a 0.1 M KCl solution [21]. The DC potential at 0 V and 10 mV was set as the peak-to-peak amplitude of the AC potentials superimposed on the aforementioned DC potential. Figure 3B depicts the Nyquist plots that were produced at frequencies ranging from 100 kHz to 0.1 Hz with an amplitude of 10 mV for the bare GCE, Ag-ZnO/GCE, MWCNTs/GCE, and MWCNTs/Ag-ZnO/GCE. The diameter of the semicircle in the Nyquist plot between the imaginary impedance (Z′′) versus real impedance (Z′) represents the resistance to charge transfer (R_ct_), while the linear part in the lower frequency region arises from diffusion limited processes characterized by Warburg impedance (W_d_) [22,23]. The semicircular section at a greater frequency corresponds to charge transfer resistance. A semicircle of smaller diameter represents lower R_ct_, and vice versa [24]. Table S2 shows R_ct_ values of 8173, 4277, 2627 and 1610 Ω for bare, Ag-ZnO-, MWCNTs- and MWCNTs/Ag-ZnO-modified GCEs, respectively. It can be seen that R_ct_ values decreased for modified electrodes as the surface area of the electrodes increased. The increase in the surface area of the electrode owing to adsorbed molecules of the MWCNTs/Ag-ZnO/GCE and its mediator role between the transducer GCE and the redox probe caused the reduction in the R_ct_ value. The availability of the active sites on the GCE increased due to the presence of the Ag-ZnO and MWCNT molecules. Immobilized molecules on the GCE surface link the analyte molecules to the electrode [25]. The modifier molecules promote ease of electron transfer between the analyte and the electrode. In addition, Ag-ZnO and MWCNTs decrease interfacial charge transfer resistance. Therefore, the selected modifier renders the surface of the GCE an excellent sensing substrate for detecting the analyte. The EIS-derived parameters in Table S2 suggest that the impedance parameters have changed because of electrode modification. This indicates that the electrode surface was successfully fabricated. By modifying the electrode with the MWCNTs/Ag-ZnO/GCE, there is an increase in the electron transfer rate between the analyte and the electrode and it decreases the charge transfer resistance. The CV and EIS data are in good agreement. In CV, the MWCNTs/Ag-ZnO/GCE shows maximum current, and EIS shows minimum resistance. Therefore, the MWCNTs/Ag-ZnO/GCE was chosen as a reliable electroanalytical sensing platform for the detection of the analyte IBP.

### 2.3. Voltammetric Analysis of the Targeted Analyte

**Figure 4:**
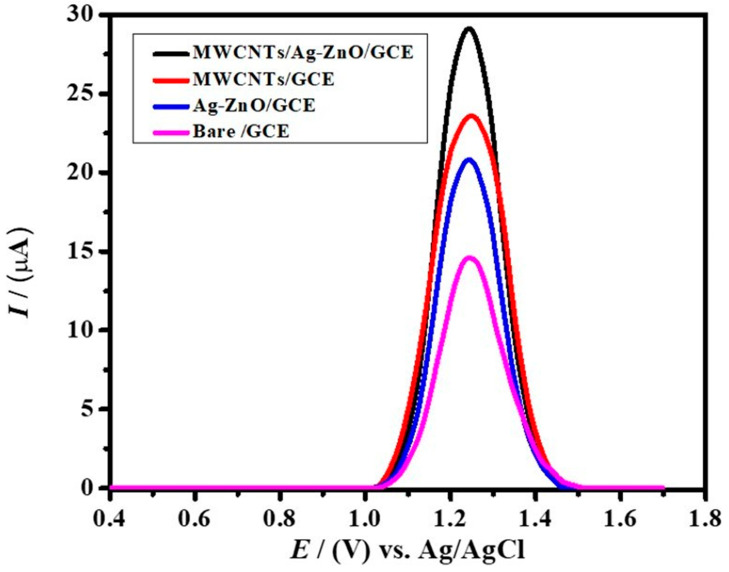
DPVs of 0.09 mM ibuprofen on bare GCE, Ag-ZnO/GCE, MWCNTS/GCE, and MWCNTs/Ag-ZnO/GCE in a supporting electrolyte of 0.9 M NaOH.

The peak current response for IBP in 0.9 M NaOH with bare and modified electrodes was studied using DPV in a potential window ranging from 0.4 V−1.7 V, as illustrated in Figure 4. The single oxidation peak depicts the oxidation of the −OH group in the IBP molecule. The modified electrode, MWCNTs/Ag-ZnO/GCE, exhibits the highest current response, with an approximately two times greater current intensity as compared to the signal for the bare GCE. The modified electrode (MWCNTs/Ag-ZnO/GCE) possesses greater catalytic performance due to the role of Ag-ZnO and MWCNTs in accelerating electron transport between the transducer and the analyte. Both MWCNTs and Ag-ZnO enhance the surface area by providing a greater number of electroactive sites to which a greater number of the analyte molecules (IBP) could be anchored. The consequent closer accessibility of IBP to the transducer led to peak current intensification at the modified electrode surface as compared to the unmodified electrode. Hence, owing to a greater number of IBP molecules on the modified interface, more molecules can be oxidized at the given potential, resulting in a greater response in terms of anodic current.

### 2.4. Effect of Scan Rate

**Figure 5:**
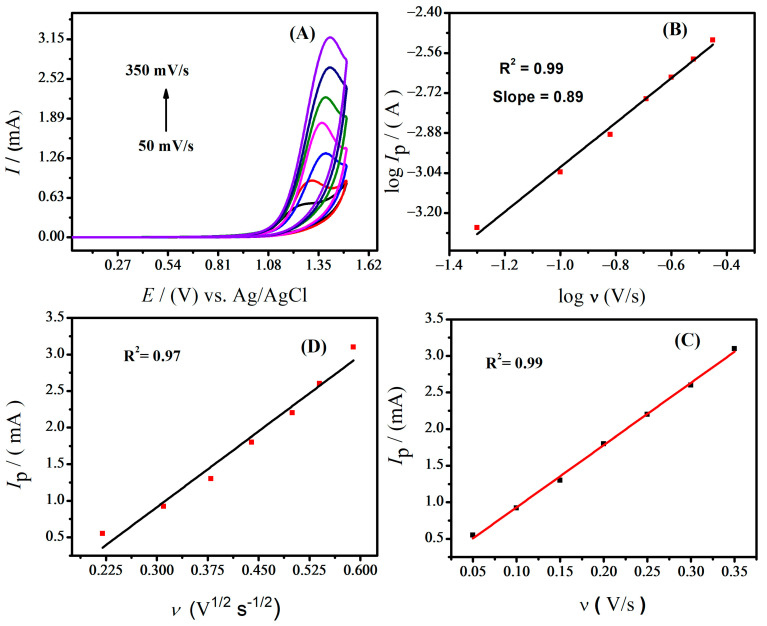
(**A**) Effect of different scan rates on the anodic peak current of IBP in supporting electrolyte (0.9 M NaOH), (**B**) calibration plot of IBP between log *I*_p_ vs. log υ, (**C**) plot of peak current vs. scan rates of IBP oxidation, and (**D**) calibration plot of IBP between peak current vs. square root of scan rates.

To analyze the effect of scan rate on the oxidation peak current of IBP, cyclic voltammograms were obtained using the MWCNTs/Ag-ZnO/GCE. At scan rates ranging from 50 to 350 mV s^−1^, cyclic voltammograms were recorded to observe the nature of the redox reaction, i.e., whether it was a diffusion or surface-controlled electrochemical process. As the scan rate increased, the peak current intensity increased proportionally (see Figure 5).

The slope value of the log of *I*_p_ and log of *ν* plot can be used to deduce the nature of the redox process. If the value of the slope is 1, the process should be controlled by adsorption; on the other hand, if the slope value is 0.5 the process should be controlled by diffusion [26]. The slope value of 0.89 as depicted in Figure 5B suggests that the electrochemical oxidation of IBP is controlled by both processes [27]. The straight-line equation for the graph shown in Figure 5B is represented by log *I*_p_ = 0.89 and log *v* − 6.57. Since the correlation coefficient in the plot of oxidation peak current vs. scan rate is higher (Figure 5C) than that of *I*_p_ vs. *ν***^½^** (Figure 5D), the process of adsorption works better on the electrode’s surface.

### 2.5. Optimization of Experimental Parameters

Among all the voltammetric techniques, DPV is the most sensitive pulse technique. Its detection limit is comparable to that achieved by chromatographic and spectroscopic approaches. After the sensing ability of the modified GCEs was tested with EIS, CV, and DPV, and the maximum current response was measured, the DPV method was used to find the best combination of experimental parameters to obtain the maximum current response. The following sections provide more information on these parameters.

#### 2.5.1. Supporting Electrolyte Optimization

Supporting electrolytes reduce the electrical potential gradient, which removes migration in a signal and minimizes the ohmic drop effect. Alternately, it increases the conductance of the solution. Supporting electrolytes impact the peak shape, position (potential), and intensity. The analyte (IBP) was tested in various supporting electrolytes to obtain optimized results. Different electrolytes were used, such as CH_3_COOH, H_2_SO_4_, NaCl, NaOH, KCl, acetate buffer (pH = 7), BRB (pH = 7), PBS (pH = 7), and KOH. The highest current response (30 μA) and the clearest definition of peak form was noticed in the solution containing sodium hydroxide as the supporting electrolyte in comparison to other electrolytes, as can be seen in Figure S2A. The high electrical conductance in NaOH may be attributed to its high solubility in water as compared to the other electrolytes investigated in this work. Moreover, it does not produce any gases and as such its concentration remains constant over time. Therefore, NaOH was selected for further electrochemical investigations.

#### 2.5.2. Effect of Accumulation Potential

An optimized accumulation potential with a value lower than the analyte’s oxidation potential during the potential sweep helps in accumulation of most of the analyte’s molecules on the surface of the electrode. This results in intense oxidation signal generation during anodic potential scanning. Hence, the impact of accumulation potential on the oxidation peak current for IBP was assessed using anodic stripping DPV. The deposition potential was in the range of −1.6 to 0.3 V. The peak current of the analyte was enhanced with the increment of deposition potential up to −1.2 V as depicted in Figure S3A. Therefore, −1.2 V deposition potential was selected for further electrochemical investigations of IBP (see Figure S3B). It is speculated that all available active sites become saturated with IBP molecules at the accumulation potential of −1.2 V and a further increase in the accumulation potential may disturb the proper orientation of IBP molecules at the electrode–electrolyte interface, thus resulting in a decrease in the current response.

#### 2.5.3. Influence of Accumulation Time

The performance of the designed electrochemical scaffold can be improved by optimizing the deposition time to obtain an enhanced peak current. Under optimized deposition potential conditions, accumulation time was varied in the range of 5 s to 50 s. IBP displayed a maximum current intensity at an accumulation time of 30 s (see Figure S4A). As the number of accessible active sites on an electrode surface increases, the peak current intensity continues to rise with an increase in the amount of time spent in depositing material. A greater concentration of analyte is accumulated on its surface. The largest peak current is seen at the point of saturation, which occurs when the analyte has become oriented to all of the available active sites. The molecules of the analyte need to be aligned in the right direction for the best possible deposition. The reduction of drug molecules occurs when they are accumulated on the electrode surface. These molecules undergo an oxidation process in the anodic stripping differential pulse voltammograms when the potential is varied from negative to positive values. At a deposition time of 30 s, maximum peak current is observed for IBP. The peak current intensity of the analyte is negatively influenced by further increasing the deposition time, as evidenced in Figure S4B.

### 2.6. Limit of Detection of IBP and Calibration Plot

**Figure 6:**
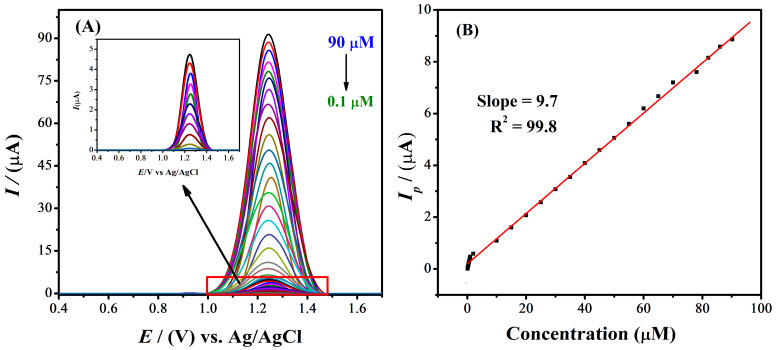
(**A**) Differential pulse voltammograms for different concentrations of IBP using an MWCNTs/Ag-ZnO/GCE under pre-optimized conditions at a scan rate of 10 mVs^−1^, (**B**) calibration plot obtained by DPV data for various concentrations of IBP.

DPV was performed to examine the LOD of IBP under optimized conditions, i.e., 0.9 M NaOH, −1.2 V accumulation potential, and 30 s deposition time. Figure 6A demonstrates that the concentration of the analyte influences the peak current. Using DPV, analyte solutions of different concentrations were tested to locate the sensor’s absolute minimum sensitivity. Figure 6B shows the electrochemical current response of the analyte at a variety of concentrations. The linear calibration curve was obtained between 0.1 to 90 µM analyte concentration. The limit of detection was calculated using the formula 3 σ/m, where m is the slope of the plot of peak current versus concentration and σ is the standard deviation of the blank signal. To compute the standard deviation, currents of the blank solution at the peak point were utilized. The LOD for the designed sensor MWCNTs/Ag-ZnO/GCE was found to be 28 nM for IBP.

One particularly interesting aspect of the developed sensor is its ability to function over a wide linearity range, from about 0.1 µM to around 90 µM (Figure 6B). It can be seen from Table 1 that the LOD value of 28 nM is significantly lower than the previously reported data for the different designed sensors. Therefore, it can be concluded that the modified electrode is a promising platform for the analytical detection of IBP.

#### 2.6.1. Estimation of the Stability of the Designed Sensor

**Figure 7:**
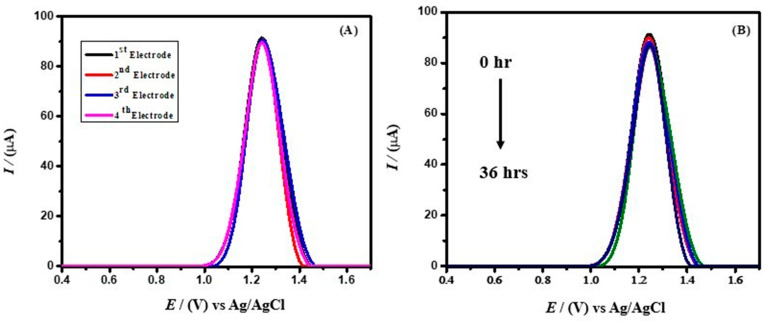
(**A**) DPVs of IBP showing reproducibility of the designed sensor using supporting electrolyte NaOH (**B**) DPVs of IBP for the designed sensor at different time intervals.

The repeatability and reproducibility of the established sensing platform were used to evaluate its stability and practical applicability. The electrochemical response of the sensor in the presence of IBP under pre-optimized testing conditions was used to evaluate the sensor stability. DPV analysis was performed on the MWCNTs/Ag-ZnO/GCE after it had been placed in NaOH for various amounts of time to assess its stability. Peak current intensity did not significantly vary (< %) with signal intensity on the newly modified electrode up to 36 h, as shown in Figure 7A. The sensing platform showed intra-day and inter-day stability of response in terms of current, remaining similar up to 36 h. Due to the poor water solubility of the components of electrode modifiers, the developed sensor showed stability over a range of time intervals. This not only prevented the modifier from eroding from the electrode surface but also kept the peak current of the analyte stable over time. Four separate MWCNTs/Ag-ZnO/GCEs were prepared and then subjected to DPV analysis for a reproducibility check of the designed sensor. The results displayed in terms of the oxidation peak show no significant deviation, asserting outstanding repeatability and reproducibility, as illustrated in Figure 7B. The percent RSD reproducibility of 0.67 and percent RSD repeatability of 1.05 are influential figures of merit for the designed sensing platform.

#### 2.6.2. Effects of Interferents for Validation of the Designed Sensor

**Figure 8:**
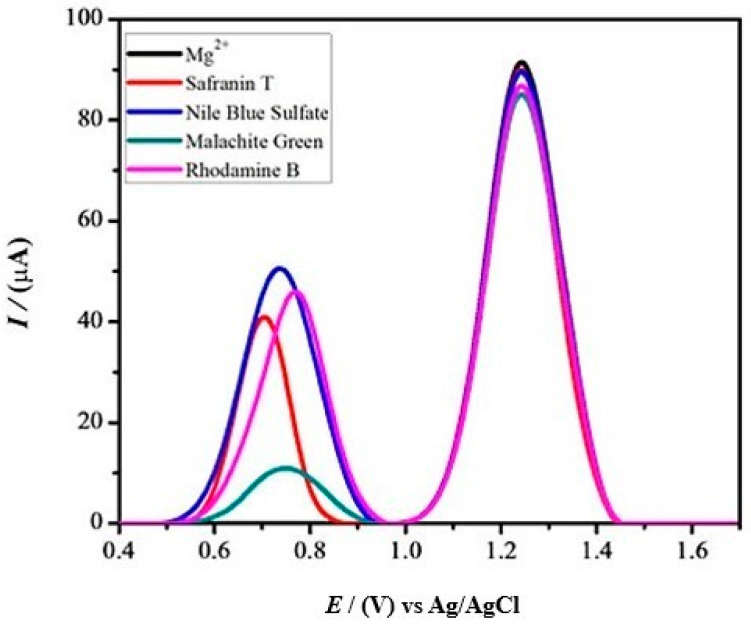
Differential pulse voltammograms for 90 μM IBP in the presence of 1 mM different interfering agents.

A real sample collected from a living being or a waste disposal site of a pharmaceutical industry or hospital may be composed of chemical species other than ibuprofen that may serve as potential interferents in the detection of the said analyte. To mimic the potential effect of the interferents, a number of chemical species, including metal ions and essential textile dyes, were individually spiked at 1 mM concentration. The voltammetric responses in the presence of interferents suggest that the IBP current signal at the designed sensor is not significantly influenced, which shows that the designed platform possesses discrimination ability for the target analyte (Figure 8).

### 2.7. Interaction Studies of IBP with BSA

Prostaglandins (PGs) are a group of lipids produced in areas of tissue damage and infection and are associated with the sensation of pain, fever, and inflammation. IBP lowers the level of PG in the body by inhibiting the cyclooxygenase enzyme, which is required for the synthesis of PG and hence can reduce pain and inflammation by lowering the PG level. Considering this effect of IBP, we evaluated the binding of IBP with bovine serum albumin (BSA, an enzyme required for synthesis of PG) using DPV. Traditionally, equilibrium dialysis has been used to evaluate the binding constants of a drug to plasma proteins. However, this method has several shortcomings, including lengthy equilibration times, usually 12–48 h. The requirement for initial studies to determine the time in which the system attains equilibrium has prompted the researchers to develop alternate techniques [37,38].

**Figure 9:**
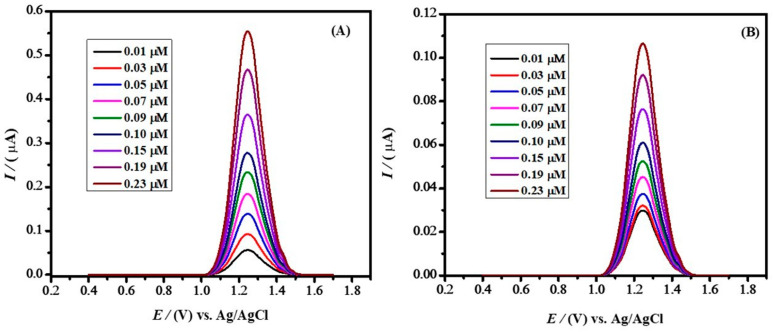
(**A**) DPV of IBP recorded with a supporting electrolyte of 0.9 M NaOH at different concentrations in the absence of BSA (**B**) DPV of IBP recorded with a supporting electrolyte of 0.9 M NaOH at different concentrations in the presence of 1 mM BSA.

For interaction studies of IBP with BSA, DPV is superior to linear scan voltammetry because of its sensitivity and ability to reduce the comparatively high background currents caused by the presence of albumin in solution [39]. For binding constant determination, all the values for peak current and its punctual difference, free drug concentration, binding constant, stoichiometry, etc., were repeated multiple times and reported values represent their means (with RSD±10% of the given values). DPV was carried out for various concentrations of IBP in the presence of 0.9 M NaOH in a potential window ranging from 0.4 V to 1.7 V at a scan rate of 10 mV s^−1^ and step potential of 5 mV (Figure 9A). IBP concentration was varied, and with decreasing concentrations peak current decreased as expected. The voltammograms were first obtained in the absence of BSA. Then, BSA in large excess (1 mM) was added to a solution of the drug, along with 0.9 M NaOH as supporting electrolyte. Figure 9B depicts the voltammograms obtained in the presence of BSA. The peak current was significantly decreased compared to the peak current of voltammograms obtained in the absence of BSA. For example, at 0.23 μM IBP, the addition of BSA decreased the current from 0.55 μA to 0.09 μA. The decreasing peak current indicates interaction between IBP and BSA, leaving less free IBP in solution for electrochemical oxidation at the electrode. A maximum decrease in peak current occurs when the maximum amount of the drug reacts with protein. Hence, the decrease in peak current is attributed to the interaction between the drug and BSA.

From the differential pulse, voltammograms recorded in Figure 9A,B show current values in the absence and presence of BSA; their punctual differences were calculated as listed in Table S3.

**Figure 10:**
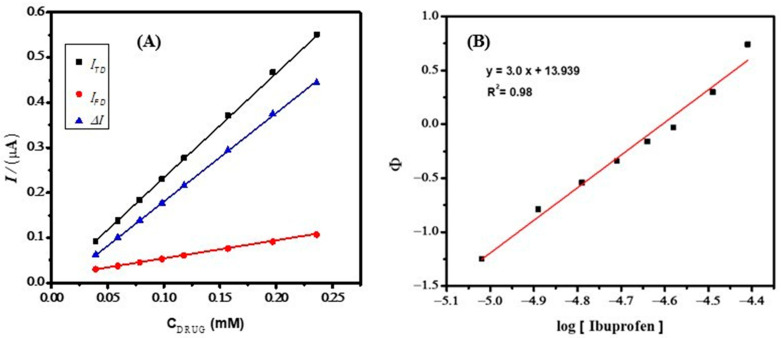
(**A**) Relationship of peak current vs. drug concentration for IBP and no BSA, NaOH 0.9 M, and 5µM BSA + NaOH 0.9 M, and punctual difference between the values of *I*_TD_ and *I*_FD_ (Δ*I* = *I*_TD_ − *I*_FD_) (**B**) relationship between Φ and log [FD] in the case of IBP with BSA, an example of a formation with a single type of complex.

The voltammetric calibration curve of IBP in 0.9 M NaOH was registered with IBP concentrations (C_drug_) in the range of 0.01 to 0.23 μM (Figure 10A). The resulting linear plot *I*_TD_ vs. C_drug_ was obtained, where *I*_TD_ represents current due to the total amount of the drug (see Figure 10A). In the same way, a voltammetric calibration plot with the same amounts of the drug (C_drug_) and a fixed amount of albumin was recorded ([ALB]total), operated with an albumin concentration of 1 mM. The resulting linear plot *I*_FD_ vs. C_drug_ was obtained, where *I*_FD_ represents the current of the free drug. For any point on the calibration curve, we may calculate the difference between the two values at that point (Δ*I* = *I*_TD_ − *I*_FD_), because the complex is not electroactive under operating conditions.

The concentration of free drug [FD] was evaluated by considering the calibration plot obtained in the absence of BSA with data given in Table S4. By plotting all values of log [FD] vs Φ, a linear plot was obtained (Figure 10B) according to equation 6 mentioned in the introduction. The value of m determined from the slope shows the number of drug molecules interacting per single molecule of BSA. The binding constant ($\beta_{s}$) with a value of 8.7 × 10^13^ was evaluated from the antilog of the intercept. A comparison of the binding constant of IBP−BSA and its stoichiometry with reported values is given in Table 2. A reasonably stronger binding of IBP with BSA is required to inhibit the functioning of BSA. The comparison of binding constant values for various protein–drug complexes shows that IBP−BSA has the highest value of binding constant, thus indicating effective inhibition of BSA by IBP.

## 3. Conclusions

A quick-responding, sensitive, and stable electrochemical sensor was developed using a composite of MWCNTs and Ag-doped ZnO nanoparticles as a modifier of a GCE surface. The designed sensor demonstrated excellent ability to detect IBP down to 28 nM. The peak current response of IBP was greatly improved by the components of the recognition layer in comparison to the bare GCE. Cyclic voltammetric and electrochemical impedance spectroscopic investigations revealed that the designed sensing scaffold has 4.5 times greater electroactive surface area and approximately 5 times less interfacial charge transfer resistance as compared to the bare GCE, which results in the generation of an intense signal for IBP oxidation. Voltammetric analysis revealed that the modified electrode possesses inter-day durability and four individually fabricated electrodes exhibited unaltered efficiency in terms of repeatability. The PG inhibition behavior of IBP was also investigated by measuring its binding capacity with BSA using DPV, where it was revealed that three molecules of IBP bind to a single molecule of BSA with a binding constant value of 8.7×10^13^. This strong binding potency of IBP suggests that it can play a significant role in PG inhibition and in turn can be developed as a medicine for reducing pain and inflammation. Considering the importance of IBP in the pharmaceutical industry and its medical potential, new and innovative analytical methods with high efficiency are still needed to effectively control this non-steroidal anti-inflammatory drug in pharmaceutical doses and to detect it in biological fluids. Moreover, coupling such sensors with industries for the early sensing of IBP in industrial effluents before their release to freshwater bodies may broaden their future applicability.

## 4. Experimental Section

### 4.1. Materials and Methods

IBP was obtained from a bio-lab pharmaceutical company (Islamabad, Pakistan) and was used as received. BSA and MWCNTs (purity > 95%) were obtained from Sigma Aldrich. CH_3_COOH, H_2_SO_4_, NaCl, NaOH, KCl, acetate buffer, Britton–Robinson buffer (BRB), phosphate-buffered saline (PBS), and KOH were tested as supporting electrolytes. Zinc acetate dihydrate and silver nitrate were purchased from Sigma Aldrich. PBS was prepared by dissolving a specified amount of Na_2_HPO_4_ and NaH_2_PO_4_ in distilled water and using 0.1 M HCl and 0.1 M NaOH for pH adjustment.

The nanomaterials were characterized using X-ray diffraction spectroscopy (X-ray diffractometer model Analytical 30440/60 X per PRO with copper K_α_ radiation source, scan rate of 0.01, and 2Ɵ range from 10°–80°) and scanning electron microscopy (JEOL.JAD-2300 module, Tokyo, Japan). A Metrohm multichannel Autolab (M101, PGSTAT302N, Utrecht, The Netherlands) equipped with NOVA 1.11 software and Gamry Interface 5000E potentiostat were used for electrochemical measurements. The electrochemical cell consisted of a glass cell with two layers of glass walls and a Teflon cover. The cap had five standard taper ports; three of them were used for the introduction of electrodes (the Ag/AgCl reference electrode, the working electrode, and the Pt auxiliary electrode), while the other two were used for the entrance and exit of inert gas purging.

### 4.2. Synthesis of Ag-ZnO

A hydrothermal method was employed to synthesize ZnO nanoparticles. Zinc acetate dihydrate (0.473 g) was dissolved in 25 mL ethanol and the pH of the solution was adjusted by dropwise addition of 1 M solution of NaOH. The solution was stirred for 10 min and then transferred to an autoclave and placed in the oven at a temperature of 200 °C for 6 h. The product was filtered and washed several times with ethanol to neutralize the pH, followed by drying at 80 °C. Ag-doped ZnO was prepared by following the same procedure except that a known amount of Ag precursor was added to the solution along with the zinc acetate precursor.

### 4.3. Electrode Modification and Detection Procedure

A clean glassy carbon electrode surface was obtained by rubbing it on a pad with 0.5 μm alumina slurry in a figure-eight pattern to keep the surface even. The surface was rinsed with a stream of distilled water to get rid of any unwanted particles. This process produced an impurity-free cleaned surface with a silver mirror-like finish. The cleanliness of the GCE was ensured by obtaining cyclic voltammograms in a potential window ranging from 0.4 V to 1.7 V that reflected the reproducibility of the obtained voltammograms [19].

The stock solution of IBP was prepared in a 1:1 mixture of distilled water and ethanol. First, a 5 µL droplet of MWCNTs with a concentration of 1 mg/mL was drop-casted on two separate pre-cleaned GCEs, followed by drop-casting 10 µL of Ag-ZnO; they were then subjected to drying in a vacuum oven at 50 °C. The performance of the designed sensing platform (MWCNTs/Ag-ZnO/GCE) was examined using differential pulse voltammetry (DPV) for the detection of IBP. The DPV was carried out at a step potential of 5 mV and scan rate of 10 mV/s. Electrochemical impedance spectroscopy (EIS) was employed to obtain the impedimetric results at an amplitude of 10 mV in the frequency range of 100 kHz to 0.1 Hz. Gamry software version 7.05 was used to fit an equivalent circuit to the obtained data and the results for the modified electrode were then compared with those of the bare electrode. Different experimental parameters such as deposition time, deposition potential, and pH of the medium were optimized, and the limit of detection (LOD) of IBP was obtained under optimized conditions. For the IBP–protein binding studies, varying concentrations of IBP were added to a 0.9 M solution of NaOH in the presence of an excess concentration of BSA (1 mM). The decrease in peak current of IBP was used for the quantification of the IBP−BSA binding constant.

## Figures and Tables

**Table 1:** Comparison of design sensors with reported sensors.

Sensors	Measurement Technique	Linear Range (μM)	LOD (nM)	Ref.
Pretreated GCE	SWSV	1.45–3.87	960	[28]
SD-MWCNT/GCE	FIA-AMP	10–1000	1900	[29]
Polyaniline nanofiber/GCE	DPSV	0.96–1.94	480	[30]
P(L-Asp)/GCE	SWV	1–150	220	[31]
MWCNT–CPE	DPV	2.36–242	9100	[32]
Clay-CPE	DPV	1–1000	835	[33]
HKUST-CNF	CV	4.84–29.08	100	[34]
Pd-PdO/Mt-CPE	DPV	0.01–0.9	28	[35]
AgNPs@Af-GO-MIP/GCE	DPV	1–100	8.7	[36]
MWCNTs/Ag-ZnO/GCE	DPV	0.1–90	28	This work

**Table 2:** Characteristics of drug complexation with OVA, BSA, and HAS.

Drug Complexes	Complex Stoichiometry (m)	Binding Constant	Ref.
Ketoprofen-BSA	3	2.4 × 10^9^	[40]
Ketoprofen-HAS	1	1.4 × 10^10^	[41]
Lorazepam-OVA	3	2.5 × 10^10^	[42]
Paroxetine-BSA	4	5.8 × 10^18^	[43]
Paroxetine-OVA	3	2.6 × 10^23^	[44]
Ibuprofen-BSA	3	8.7 × 10^13^	This work

## Data Availability

The data presented in this study are available within the article and supplementary material.

## Supplementary Materials

The following supporting information can be downloaded at: https://www.mdpi.com/article/10.3390/molecules28010049/s1, Figure S1: Source of ibuprofen pollution.; Table S1: Electroactive surface areas of bare and modified electrodes; Table S2: EIS-derived parameters. Solution resistance (R_s_), Charge transfer resistance (R_ct_), constant phase element (CPE). Figure S2: (A) Effect of various supporting electrolytes on the anodic peak current of IBP using MWCNTs/Ag-ZnO modified GCE (B) Bar graph of IBP between peak current vs. various supporting electrolytes; Figure S3: (A) Effect of deposition potential on the peak current of 0.09 mM ibuprofen in NaOH using MWCNTs/Ag-ZnO/GCE at 30 s deposition time. (B) Plot of *I*_p_ vs. deposition potential; Figure S4: (A) Effect of deposition time on the peak current of 0.09 mM ibuprofen using MWNTs/Ag-ZnO/GCE. (B) Plot between Ip vs. accumulation time; Table S3: The values of current for total drug (*I*_TD_) free drug (*I*_FD_) and the difference of the two currents Δ*I*; Table S4: Data employed for the determination of function Φ and for the construction of plot Φ versus log [FD] in the case of the IBP-BSA complex in Figure 10.

## Sample Availability

Samples of the compounds are available from the authors.
