# Supplementary material for: Electroanalysis of Ibuprofen and Its Interaction with Bovine Serum Albumin
Source: Molecules. 2022 Dec 21;28(1):49. doi: 10.3390/molecules28010049 (PMC9821973; doi:10.3390/molecules28010049)
Supplement: Supplementary file 1 [file molecules-28-00049-s001.zip › molecules-2059867-supplementary.pdf]

Electroanalysis of ibuprofen and its interaction with bovine serum albumin

Muhammad Dilshad [1], Afzal Shah [1,*] and Shamsa Munir [2]

[1] Department of Chemistry, Quaid-Azam University, Islamabad 45320, Pakistan
[2] School of Applied Sciences and Humanities, National University of Technology (NUTECH), Islamabad 44000, Pakistan
* Correspondence: afzals_qau@yahoo.com and afzalshah@qau.edu.pk

Supplementary Information

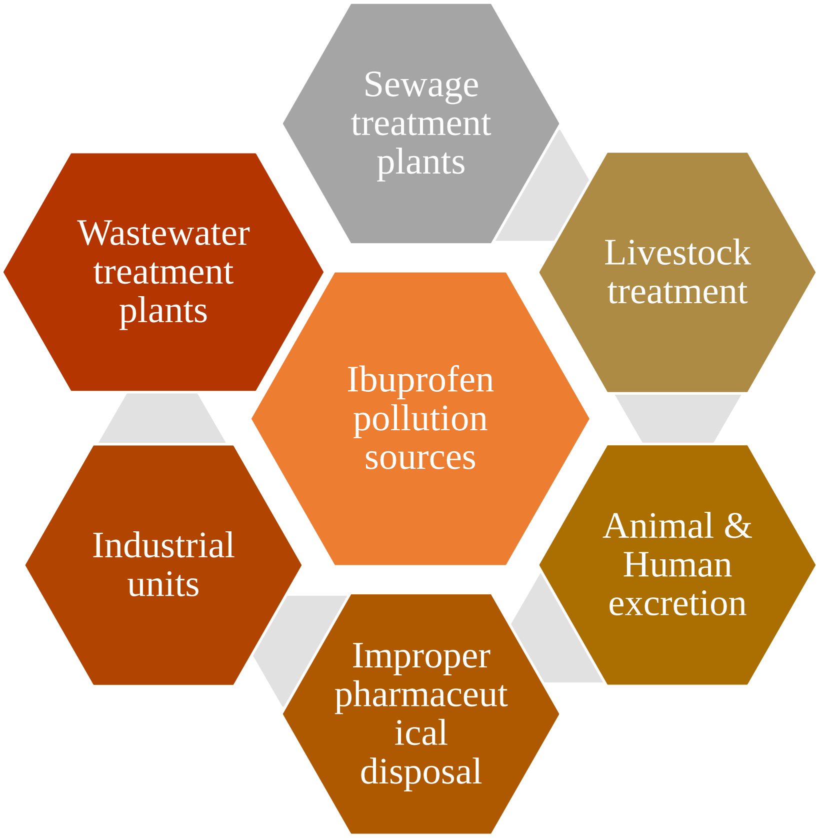

Figure S1. Source of ibuprofen pollution.

Calculation for multiple type of complex formation

In case of multiple type of complex formation, a mathematical expression is obtained as follows.

$$\Delta I = \frac{\Delta I_1 \beta_1 [FD] + \Delta I_2 \beta_2 [FD]^2 + \cdots + \Delta I_n \beta_n [FD]^n}{1 + \beta_1 [FD] + \beta_2 [FD]^2 + \cdots + \beta_n [FD]^n}$$

Where ΔI is the total decrease of peak current I_p obtained through a current voltage measurement.

$$f_1 = \frac{\Delta I}{[\,FD\,]}$$

$$f_1 == \frac{\Delta I_1 \beta_1 + \Delta I_2 \beta_2 [FD]^1 + \cdots + \Delta I_n \beta_n [FD]^{n-1}}{1 + \beta_1 [FD] + \beta_2 [FD]^2 + \cdots + \beta_n [FD]^n}$$

Tangent (f_1 vs [FD]) the curve intersects the x-axis at a_1 and has a slope a_2 when $[FD] \rightarrow 0$
If x represents the concentration of free drug then in the limit when x when approaches to zero.

$$\lim_{x \to 0} f_1 = \Delta I_1 \beta_1 = a_1$$

By taking a plot of f_1 as a function of $[FD]$:

$$\lim_{x \to 0} \frac{df_1}{d[FD]} = \Delta I_2 \beta_2 - \Delta I_1 \beta_1^2 = a_2 \text{ , where denoting } \frac{1}{[\,FD\,]} \text{ by Z}$$

and multiplying the above equation by Z^n/Z^n yields the following equation

$$\Delta I = \frac{\Delta I_n \beta_n + \Delta I_{n-1} \beta_{n-1} Z + \cdots + \Delta I_1 \beta_1 Z^{n-1}}{\beta_n + \beta_{n-1} Z + \cdots + \beta_1 Z^{n-1} + Z^n}$$

$$\lim_{x \to 0} f_1 = \Delta I = \Delta I_n = b_1$$

$$\lim_{x \to 0} \frac{d\Delta I_1}{dZ} = \frac{(\Delta I_{n-1} - \Delta I_n)\beta_{n-1}}{\beta_n} = b_2$$

$$\Delta I_1 \beta_1 = a_1$$

$$\Delta I_1 = \frac{a_1}{\beta_1}$$

$$\Delta I_2 \beta_2 - \Delta I_1 \beta_1^2 = a_2$$

As

$$\Delta I_2 = b_1$$

$$\frac{(\Delta I_1 - \Delta I_2)\beta_1}{\beta_2} = b_2$$

So, by putting $\Delta I_1 = \frac{a_1}{\beta_1}$ and $\Delta I_2 = b_1$ and by solving,

$$\frac{\left(\frac{a_1}{\beta_1} - b_1\right)\beta_1}{\beta_2} = b_2$$

$$\frac{(a_1 - b_1\beta_1)}{\beta_1} \times \frac{\beta_1}{b_2} = \beta_2$$

$$\frac{(a_1 - b_1\beta_1)}{b_2} = \beta_2 \frac{(a_2 + a_1\beta_1)}{b_1} \times \frac{(a_1 - b_1\beta_1)}{b_2}$$

$$a_2 b_2 + a_1\beta_1 b_2 = a_1 b_1 - b_1^2 \beta_1$$

$$a_1\beta_1 b_2 + b_1^2\beta_1 = a_1 b_1 - a_2 b_2$$

$$\beta_1(a_1 b_2 + b_1^2) = a_1 b_1 - a_2 b_2$$

$$\beta_1 = \frac{a_1 b_1 - a_2 b_2}{a_1 b_2 + b_1^2}$$

$$\beta_2 = \frac{1}{b_1}\left(a_2 + a_1\left(\frac{a_1 b_1 - a_2 b_2}{a_1 b_2 + b_1^2}\right)\right)$$

$$\beta_2 = \frac{1}{b_1}\left(a_2 + \left(\frac{a_1^2 b_1 - a_1 a_2 b_2}{a_1 b_2 + b_1^2}\right)\right)$$

$$\beta_2 = \frac{1}{b_1}\left(\frac{a_2(a_1 b_2 + b_1^2) + a_1^2 b_1 - a_1 a_2 b_2}{a_1 b_2 + b_1^2}\right)$$

$$\beta_2 = \frac{1}{b_1}\left(\frac{a_2 a_1 b_2 + a_2 b_1^2 + a_1^2 b_1 - a_1 a_2 b_2}{a_1 b_2 + b_1^2}\right)$$

$$\beta_2 = \frac{1}{b_1}\left(\frac{a_2 b_1^2 + a_1^2 b_1}{a_1 b_2 + b_1^2}\right)$$

$$\beta_2 = \frac{b_1}{b_1}\left(\frac{a_2 b_1 + a_1^2}{a_1 b_2 + b_1^2}\right)$$

$$\beta_2 = \frac{a_2 b_1 + a_1^2}{a_1 b_2 + b_1^2}$$

$$\beta_1 = \frac{a_1 b_1 - a_2 b_2}{a_1 b_2 + b_1^2}$$

$$\beta_2 = \frac{a_2 b_1 + a_1^2}{a_1 b_2 + b_1^2}$$

Table S1. Electroactive surface areas of bare and modified electrodes.

**Working Electrode**	**Surface Area (cm^2)**
Bare GCE	0.02
Ag-ZnO /GCE	0.05
MWCNTs /GCE	0.06
MWCNTs/Ag-ZnO/GCE	0.09

Table S2. EIS-derived parameters. Solution resistance (R_s), Charge transfer resistance (R_{ct}), constant phase element (CPE).

**Working Electrode**	**R_s (Ohm)**	**R_{ct} (Ohm)**	**CPE (μF)**
Bare GCE	125.4	8173	47.2
Ag-ZnO/GCE	126.6	4277	82.7
MWCNTs/GCE	123.3	2627	1.5
MWCNTs/Ag-ZnO/GCE	124.9	1610	1.2

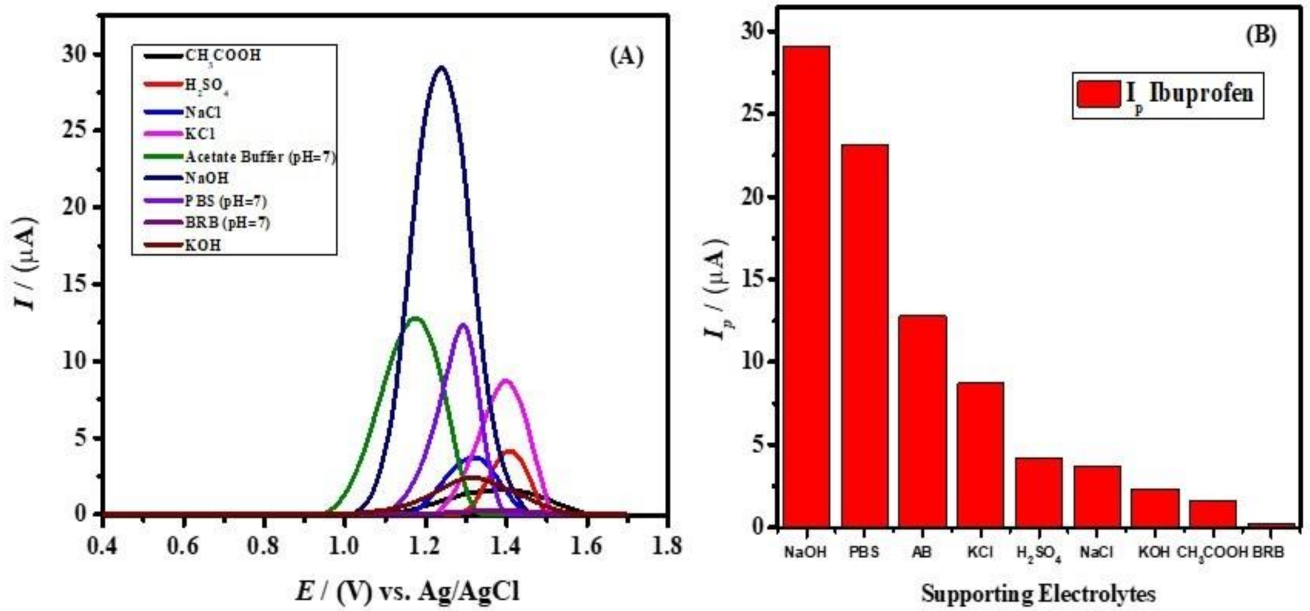

Figure S2. (A) Effect of various supporting electrolytes on the anodic peak current of IBP using MWCNTs/ Ag-ZnO modified GCE. (B) Bar graph of IBP between peak current vs. various supporting electrolytes.

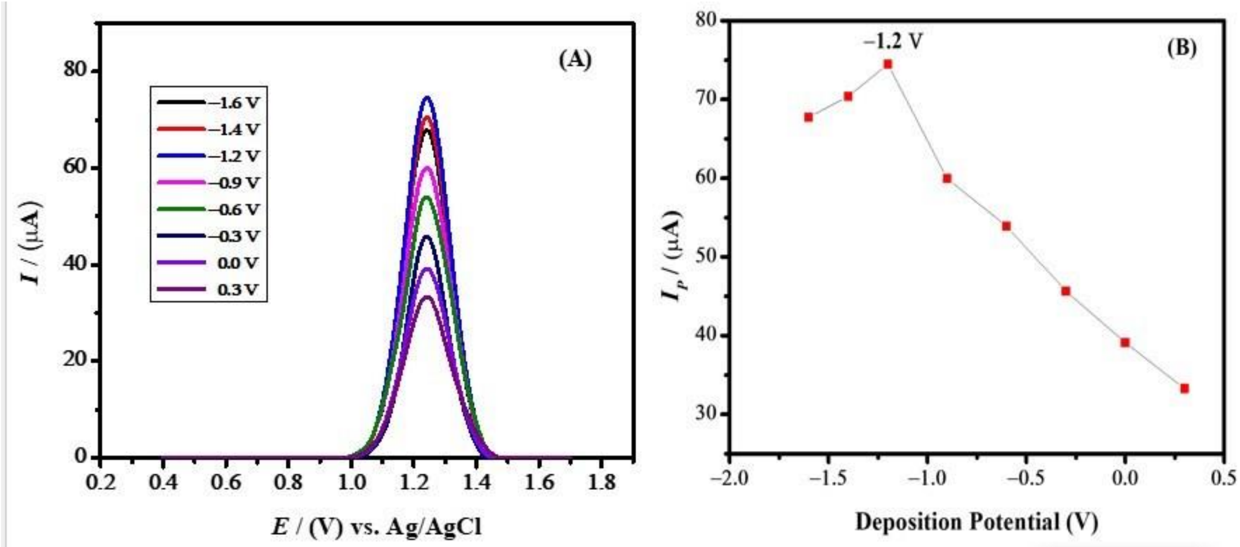

Figure S3. (A) Effect of deposition potential on the peak current of 0.09 mM ibuprofen in NaOH using MWCNTs/Ag-ZnO/GCE at 30 s deposition time. **(B)** Plot of I_p vs. deposition potential.

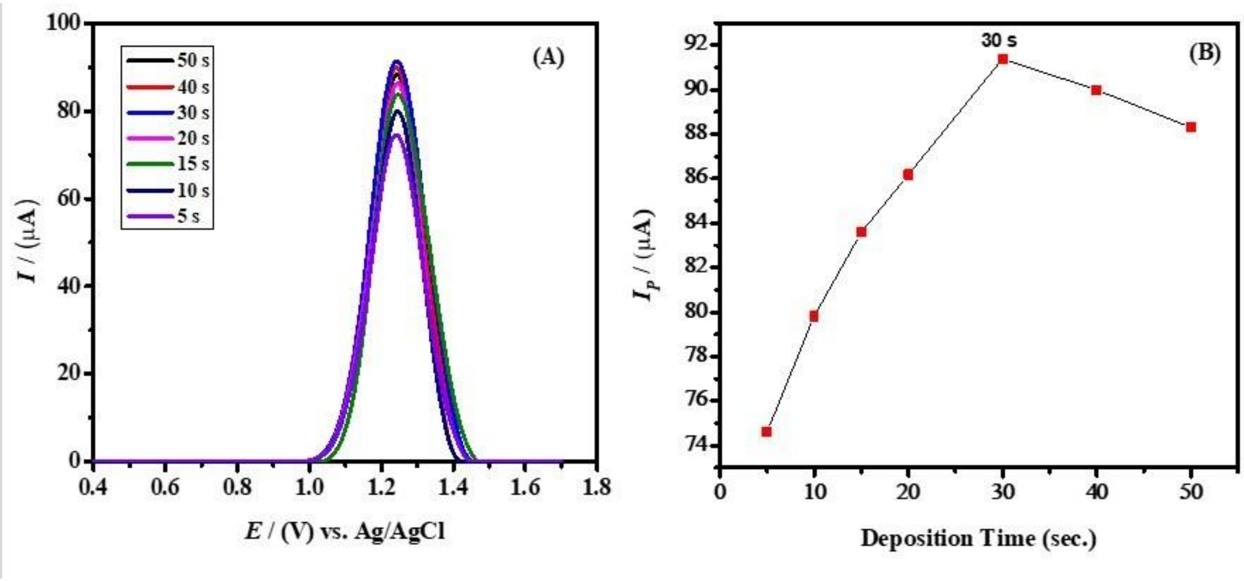

Figure S4. (A) Effect of deposition time on the peak current of 0.09 mM ibuprofen using MWNTs/Ag-ZnO/GCE. (**B)** Plot between Ip vs. accumulation time.

Table S3. The values of current for total drug *(*I_{TD}) free drug *(*I_{FD}*)* and the difference of the two currents Δ*I*.

**Ibuprofen C_{drug} (μM)**	**I_{TD} (μA)**	**I_{FD} (μA)**	**ΔI= I_{TD} - I_{FD} (μA)**
**0.01**	0.06	0.022	0.038
**0.03**	0.09	0.03	0.060
**0.05**	0.14	0.037	0.100
**0.07**	0.18	0.045	0.135
**0.09**	0.23	0.052	0.178
**0.1**	0.27	0.060	0.210
**0.15**	0.37	0.076	0.294
**0.19**	0.46	0.091	0.369
**0.23**	0.6	0.01	0.59

Table S4. Data employed for the determination of function Φ and for the construction of plot Φ versus log [FD] in the case of the IBP-BSA complex in Figure 10.

C_{drug} (μM)	[FD][a] (M)	Log [FD] (M)	Δ*I* (A)	Φ
**0.01**	9.5×10^{-06}	-5.02	0.038	-1.25
**0.03**	1.28×10^{-5}	-4.89	0.060	-0.79
**0.05**	1.61×10^{-5}	-4.79	0.100	-0.54
**0.07**	1.94×10^{-5}	-4.71	0.135	-0.34
**0.09**	2.27×10^{-5}	-4.64	0.178	-0.16
**0.1**	2.65×10^{-5}	-4.57	0.210	-0.03
**0.15**	3.27×10^{-5}	-4.48	0.294	0.3
**0.19**	3.95×10^{-5}	-4.40	0.369	0.74
**0.23**	4.59×10^{-5}	-4.34	0.59 (ΔI_{max})	-
